# The Story of Equine Atypical Myopathy: A Review from the Beginning to a Possible End

**DOI:** 10.5402/2012/281018

**Published:** 2012-11-14

**Authors:** Dominique-Marie Votion

**Affiliations:** Equine Clinic, Faculty of Veterinary Medicine, University of Liege, Boulevard de Colonster 20 B41, 4000 Liege, Belgium

## Abstract

Atypical myopathy (AM) is a frequently fatal seasonal pasture myopathy that emerges in Europe. Outbreaks are of an acute and unexpected nature and practitioners should be prepared to handle these critically ill patients. This review retraces the history of AM and describes results of epidemiological investigations that were conducted to raise hypotheses concerning the etiology of this devastating disease as well as to be able to suggest potential preventive measures. Also, clinical studies have contributed to a better definition and recognition of the syndrome, whereas elucidation of the pathological process, identified as a multiple acyl-CoA dehydrogenase deficiency (MADD), was a great step forward improving medical management of AM and guiding the search for the etiological agent towards toxins that reproduce the identified defect. Treatment plans can be extrapolated from the described clinical signs and metabolic problems, but they remain limited to supportive care until the causative agent has been identified with certainty. Since treatment is still unsuccessful in the majority of cases, the main emphasis is currently still on prevention. This paper aims at being a practical support for equine clinicians dealing with AM and is based on discussion and comparison of the currently available scientific data.

## 1. From the Beginning

An apparently new equine myopathic syndrome was described in a special report of a meeting attended by practitioners and scientists that was published in January 1985 following autumnal outbreaks in Great Britain [1]. The syndrome was named atypical myoglobinuria and its main features were summarized. Most of these features have been confirmed over the years, such as the sudden onset of stiffness, muscular weakness, tachycardia, a preserve appetite, and the emission of dark chocolate urine [2, 3]. Also, the syndrome was atypical because, as opposed to exercise-induced rhabdomyolysis syndromes, the animals affected were kept at grass with limited supplementary feed, the myopathy was not induced by exertion, and most of affected horses died within a couple of days. Most probably, the syndrome was already encountered in the past since reports of outbreaks of myopathy in grazing horses that occurred in the fall are found in the literature in the early forties [4] and in succeeding years [5–7]. Since its recognition as a specific condition [1], case reports have sporadically been reported in the literature [8–12]. Later on, atypical myoglobinuria was renamed as atypical myopathy (AM) to refer to the underlying pathological process (i.e., a myopathy occurring in the above-mentioned atypical conditions when compared to exercise-induced rhabdomyolysis syndromes) rather than to one of its possible clinical signs, that is, myoglobinuria [13–15], that is not present in all cases (*see further*) [2, 3].

The first large outbreak (i.e., more than one hundred horses died of the condition) occurred in the fall of 1995 in Northern Germany where the syndrome had not been diagnosed before [16]. Since the new millennium, this highly fatal pasture myopathy appears to emerge in Europe and now the disease has been recognized in Belgium, Denmark, France, Germany, Ireland, United Kingdom, Latvia, Luxembourg, Spain, Switzerland, and The Netherlands [2, 3, 13, 15–21] but also in Austria, Czech Republic, Italy, Norway, and Sweden (unpublished data from the Atypical Myopathy Alert Group; AMAG). In Belgium, Germany, and France large outbreaks of AM are now encountered at regular intervals and they are the European countries with the most reported cases [2].

Also, outbreaks of acute nonexertional pasture-associated rhabdomyolysis that strongly resemble AM were described in Canada [6] and in the United States of America (USA) [7, 22]; the USA syndrome was called seasonal pasture myopathy (SPM). It is now believed that SPM is indeed similar to AM as it has been showed that biochemical defects of both syndromes are identical [23, 24]. Cases compatible with a diagnosis of AM also have been reported from Australia [25] and New Zealand (information from AMAG).

With the increasing number of cases and outbreaks, and the recurrence of outbreaks, epidemiological investigation appeared as a necessity to raise hypotheses concerning the etiology of this devastating disease as well as to be able to suggest potential preventive measures. Also, collection of precise clinical data was needed to refine the syndrome description and hopefully to improve the clinical management of this often fatal condition [26]. In 2004, epidemiological and clinical questionnaires were designed to collect the required data for a consensual description of the syndrome and for risk analysis [14]. The University of Liege established an informal epidemiosurveillance network called AMAG [27] that, in 2006, expanded the recording of national cases to European cases. Currently, this network consists of worldwide equine veterinarians, national epidemiological networks, and universities. Its purpose is to exchange information about the occurrence of outbreaks of this disease and to initiate collaborative researches. Since the recording of European cases, more than a thousand potential AM cases have been communicated to the AMAG (see Table 1). Epidemiological and clinical data from these cases and outbreaks have contributed to the identification of indicators or factors related to the individual horse, managemental practices, and pasture characteristics that are associated to AM [2, 3]. With these data the diagnosis of AM was improved, prognostic predictors were identified, risk and protective factors were identified, and preventive measures were recommended [28, 29]. Also, epidemiological investigations together with the contribution of specific laboratory researches have raised some etiological hypotheses and ruled out others. The great difficulty to conduct major European research projects on AM has always been the impossibility to get a significant grant (with the exception of a two-year grant given by the “Ministère de l' Agriculture et de la Ruralité de la Région Wallonne” of Belgium to study Belgian outbreaks when the disease was first recognised in Belgium; Grant no. 2800/1/0—from 2003 to 2005) in part because of its nonzoonotic character and the targeted horse population, that is, pastured and leisure horses with limited monetary value. Therefore, most of the studies have focused on noncostly but time-consuming studies, that are, epidemiological investigations. In contrast, in the USA, a research is funded by the Minnesota Rapid Agricultural Response Fund [30] and a collaborative work has been initiated with the University of Liege which has a large library of AM cases with samples of all kinds collected over many years.

This paper reviews the literature on AM in order to gather all the available information relating to this syndrome including the symptomatic treatment and management of the disease that can be suggested based on the current knowledge of the condition. Finally, the paper discusses the etiological hypotheses that have been raised over time and ends with the latest knowledge that opens perspective for a happy ending.

## 2. History

The most remarkable feature of the disease is the *sudden onset* of clinical signs consistent with an acute, *not excercise-related* myopathic process in horses *at pasture* [2, 3, 10, 12, 13].

Horses, draft horses, and ponies of various breeds, but also donkeys and zebras, have suffered from AM [2]. The condition affects predominantly young animals [29], but horses of all ages including very old horses can be affected [2]. There is no sex predilection, but because females are more frequently kept at pasture than males they were more often affected in Belgium [29], but on European level both sexes were equally represented [2]. In general, affected horses were in good body condition even if most of them received only limited supplementary feed [2, 3, 10, 12].

Outbreaks have mostly been observed in the fall and in the spring [2, 10, 12, 13, 15, 16]. The higher incidence of cases during fall is thought to be linked to weather conditions. However, it is difficult from the literature to point out a specific climatic feature. The weather prior to the onset of outbreaks was described as ‘‘very wet and cold” [9, 12], ‘‘stormy, cold, and humid” [10], ‘‘stormy” [8], or ‘‘stormy and humid” [3, 13, 22, 31]. Moreover, Belgian outbreaks were characterized by a lack of solar radiation, an excess of precipitation or relative humidity, and no frost in the preceding days [3]. Seasonal pasture myopathy in the USA has also been associated with an absence of severe frost [22]. Whatever the common factor is, if such a factor exists, climatic conditions appear to play a role in the pathogenesis of AM, toxigenicity, and/or availability of the causal agent (e.g., by making the causal agent available for ingestion when the wind brings vegetation to the ground or by providing specific conditions required to the production of toxins). Development of new cases during an outbreak ceases after 4-5 days of heavy frost and/or snow (personal observation), which suggests that extreme cold may destroy the causative agent or render it inaccessible for the horse. It also suggests insects such as caterpillars or endophytes and/or some agents that might be controlled with mechanical or chemical means.

Although the disease does not show characteristics of a contagious disease, several horses within a group may be affected simultaneously [3, 13, 15, 16, 31], most probably because the grazed pasture presents a similar particular environment at a specific moment. Pastures on which AM occur are typically described as having a slope and containing dead leaves, dead wood, and humid zones, and containing or being surrounded by trees [2, 3, 12, 13, 31]. Clinical cases have, however, also occurred on pastures not resembling this description [2]. Pastures where cases declare are often restricted to particular regions within a country [2, 3, 12, 16, 29, 31] reinforcing the idea of a favourable environment or conditions for the causal agent to be present and/or to exert its toxicity. Botanical surveys mentioned repeatedly, but not consistently, the presence of *Acer pseudoplatanus* (maple tree; Aceraceae) in pastures that were examined with regards to AM [3, 16, 31, 32] except where the visited pastures were those of cases confirmed for AM after histological examination [3] or identification of the biochemical profile characteristic of AM (*see* [32] *for further information about the diagnosis of AM*). Although not a case-control study, a recent European descriptive study identified that 98% of the 354 described AM cases had trees in or around the pasture. In addition, a comparison of these 354 AM cases *versus* sick horses reported as AM but finally classified as having another disease recognized the presence of trees, dead leaves, and dead wood at pasture as indicators of a risk factor for development of the disease [2, 28, 29]. All these findings together suggest that the implication of trees in the development of AM should be further investigated. All cases occurred while grazing more than 6 hours per day, except some horses that were very recently stabled since up to 4 days [2, 3].

## 3. Clinical Signs

Many of the clinical signs of AM are the consequence of degeneration of postural and respiratory muscles, which are the main targets of the disease [33]. The clinical syndrome is characterized by the sudden onset of pronounced muscular weakness and stiffness and can rapidly progress: horses often develop respiratory difficulties and recumbency and the majority die within 72 hours of the onset of signs [2, 3, 10, 12, 13, 16, 31]. The emission of dark colored urine due to myoglobinuria is an indicative clinical sign; however, since it is a consequence of severe rhabdomyolysis, it can occur following other severe rhabdomyolysis syndromes. Moreover, absence of myoglobinuria does not exclude AM. Dependent on the moment of examination of the patient according the onset of clinical signs and the severity of rhabdomyolysis, myoglobinuria can be missed and/or be less pronounced [2, 3].

Affected horses can be anorexic or dysphagic, but more often they have a normal to even ravenous appetite. They can display hypothermia or have a rectal temperature within normal range, while some are hyperthermic. Tachycardia and congested mucous membranes are often present [2, 3, 10, 12, 13, 16, 22, 31]. When rectal palpation is performed, frequently a distended bladder [3, 10, 16] and sometimes dry, mucous covered feces or colon impactions [2] are encountered. These findings can lead to colic signs or discomfort [3, 31]. In general, pain is reported to be variable and mostly mild to moderate, although pain can be absent or on the contrary severe to extreme in some cases [12, 16].

Less common clinical signs that have been reported in AM are icterus, paddling, hemorrhagic diathesis, edema at the head, absence of consciousness, cardiac arrhythmias, mild diarrhea, colon displacement, perirenal pain, reduced rectal tone, penile prolapse, buccal necrosis or ulceration, ptosis, trismus, laminitis, and clinical signs consistent with renal dysfunction or congestive heart failure [2, 3, 16, 34].  Table 2 shows the frequency of the main clinical features of AM.

## 4. Pathophysiology

Histochemical staining of AM affected muscle for adenosine triphosphatase (ATPase) highlights that the degenerative process selectively targets type I rather than type II fibres [16] and indicates increased lipid storage in these fibres [17, 33]. These observations, along with absence of glycogen or polysaccharide accumulations, suggest an impaired oxidative metabolism and a preserved glycolytic pathway [33]. Also, AM exhibits biochemical changes consistent with multiple acyl-CoA dehydrogenase deficiency (MADD) [24]. MADD results, among others, from defects in several mitochondrial dehydrogenases that use flavin adenine dinucleotide (FAD) as a cofactor. Thus, this metabolic disorder impairs the mitochondrial aerobic metabolism, especially the *β*-oxidation pathway, and causes an energetic imbalance. Although MADD in humans is considered a genetic disorder [35], the cause of this lipid storage myopathy in horses probably results from a toxin that mimics this dysfunction of fatty acid oxidation (*see etiological hypotheses*). Identification of this metabolic defect was of paramount importance since it improved diagnosis and the therapeutic management of AM and guides the search for the etiological agent towards toxins that reproduce these defects.

## 5. Diagnosis of Atypical Myopathy

A recent European risk analysis compared AM-affected horses to horses that were reported to AMAG as being suspected for AM, but finally categorised as having a low likelihood of having AM or diagnosed with another condition (non-AM group) [28]. Because this comparison points out the difficulties of correctly diagnosing AM, differences between groups are not only indicators of risk factors, but are also of diagnostic aids. Variables concerning the history of cases that were significantly more often encountered in AM cases compared with non-AM cases in this study were as follows: the presence of trees, dead wood and accumulations of dead leaves at pasture, a sloping pasture, normal body condition, and full-time grazing. Variables that were more often present in non-AM cases compared with AM cases were as follows: poor body condition, water provision from a tank or bath tub, food supplementation, and spending no time or limited time at pasture.

So diagnosis of AM is not straight forward, and AM easily mimics other acute equine diseases. Differential diagnosis of AM includes other severe rhabdomyolysis syndromes amongst which: recurrent exertional rhabdomyolysis, polysaccharide storage myopathy, post-anaesthetic myopathy, immune-mediated myopathy, clostridial myonecrosis, toxic myopathy due to ingestion of ionophores or toxic plants (for example, *Eupatorium rugosum* (white snakeroot, *Compositae*) and *Cassia occidentalis* (coffee weed or coffee senna, *Caesalpiniaceae*)), nutritional myopathy, hyperkalemic periodic paralysis, malignant hyperthermia, or not yet identified lipid disorders. Other conditions with endotoxemic or hypovolemic shock must be considered (especially colic) as well as conditions leading to abnormal gait or recumbency (e.g., laminitis, neurological disease, hypocalcemia, and pleuropneumonia). In addition, the acute form of equine grass sickness is a condition that has several similarities with AM as regard to clinical signs, epidemiology, and associated risk factors [36].

Although most reported cases had not been exercised prior to the onset of acute myopathy [2, 3, 12, 13, 16, 31], some have [15]. In addition, most cases of AM were not in training or only in minimal training at the time of diagnosis [2]. When clinical signs of acute myopathy develop in relation to exertion, the diagnosis of exertional rhabdomyolysis is more likely.

The European risk analysis mentioned above compared not only historical but also clinical variables. The clinical signs that were significantly more often encountered in AM-affected horses than in the non-AM group in this study were pigmenturia, normothermia, congested mucous membranes, and high serum creatine kinase (CK) values [28]. These clinical signs may be useful to strengthen a clinical suspicion of AM, but other conditions may also lead to these signs (*see above*), whereas not all AM-affected horses show them. Indeed, any rhabdomyolysis syndrome results in myoglobinuria. However, it is worth noting that the urine of AM-affected horses is particularly dark chocolate most probably because of the extensive muscle damage associated to the condition. Generally speaking, myoglobinuria must be differentiated from hematuria and hemoglobinuria. Horses with cyanotic mucous membranes or sudden death without observation of previous clinical signs were more often categorised in the non-AM group. Other disorders, such as acute colic, probably remain a more frequent cause of sudden death and cyanosis. Although ‘‘being found dead at pasture without prodromal signs” is often associated to AM and was a major feature in a study on autopsied horses with AM being presented by 18/32 cases [33], this clinical presentation was only reported in 3/57 [3] and 2/327 [2] horses diagnosed with AM in clinical studies. It should therefore probably not be categorised as being a typical or main feature of AM.

Based on the same European AM cases and non-AM cases, sensitivity, specificity, positive, and negative predictive values (PPV and NPV, resp.) of common clinical signs were calculated related to diagnosis [28]. Pigmenturia had a high sensitivity and PPV in this study, which means that the proportion of AM cases with pigmenturia on all AM cases was high (only few AM cases had no pigmenturia) and that the proportion of AM cases with pigmenturia on all horses with pigmenturia in the study was high (only few cases with pigmenturia had another disease than AM). Normothermia and congested mucous membranes had a high PPV for diagnosis of AM, meaning that the proportion of AM cases with these signs on all horses with these signs in the study was high. It should be mentioned that PPV and NPV are related to the prevalence of the condition in the population [37], and variables with a high PPV and/or NPV should therefore not be used as diagnostic aid in a population with a low prevalence. However, in a population of horses with acute myopathic signs they might be useful as they approach the prevalence of the study population of van Galen et al. (2012) [28]. On the contrary, sensitivity and specificity are not influenced by prevalence. Mostly variables with a high sensitivity (pigmenturia) are useful for clinicians with respect to diagnosis of AM, because few false negatives are encountered when using them as diagnostic aid and are therefore ideal as a first screening tool. Although they can be another interesting piece of the diagnostic puzzle, further clinical and laboratory confirmation is of course needed.

## 6. Laboratory Findings

### 6.1. Standard Blood and Urine Analysis

The most helpful laboratory test to screen for AM is probably the determination of the serum activities of muscle enzymes and the presence of myoglobin in urine samples. A massive rhabdomyolysis can be confirmed when serum activities of CK, aspartate aminotransferase (AST), and lactate dehydrogenase (LDH) are highly increased. In most cases, CK values attain hundreds of thousands or even exceed the millions of international units per liter [2, 3, 10, 12, 13, 16, 22, 31]. However, in some cases, they may only be slightly increased when measured shortly after the onset of clinical signs, and therefore repeated analysis can be recommended [2]. Also, it is necessary to remind that recumbent horses, independent of the cause, often show mild to moderate increased muscle enzyme activity.

Acid-base imbalances (mainly lactic acidosis, measured strong ion difference [mSID], alkalosis, and respiratory disorders) are common in horses suffering from AM [38]. Also electrolyte derangements, such as hypocalcaemia and hypochloraemia, are often reported, and occasionally hypomagnesemia, hyperphosphatemia, mild hyponatremia, mild hypernatremia, and mild hyperkalemia occur [3, 13, 22, 31, 34, 38].

Other common laboratory findings are leucocytosis due to neutrophilia, hyperglycemia, hyperlipemia, and increased troponin, haptoglobin, packed cell volume (PCV), urea, creatinine, and liver enzyme activities [3, 10, 12, 13, 16, 24, 38]. The selenium status varies from normal to severely deficient [10, 12, 13, 16].

### 6.2. Acylcarnitines, Organic Acids, and Glycine Conjugates

Analysis of acylcarnitines in urine or blood and analysis of organic acids and glycine conjugates in urine allow identification of the biochemical profile of MADD [24, 39]. These laboratory tests can be performed at most human laboratories, but reference values with healthy control horses must be established. Affected horses have been demonstrated to have significant increases in urinary lactic acid, ethylmalonic acid, 2-methylsuccinic acid, butyrylglycine, (iso)valerylglycine, hexanoylglycine, free carnitine, C2-, C3-, C4-, C5-, C6-, C8-, C8:1-, C10:1-, and C10:2-carnitine. In plasma, they showed significant increases in lactic acid, C2-, C4-, C5-, C6-, C8:1-, C10:1-, and C10:2-carnitine. Plasmatic free carnitine and C3- and C8-carnitine were increased in most, but not all cases. In the same study by Westermann et al. [24], horses suffering from other acute myopathies were also examined and two out of three were shown to have increased plasmatic-free carnitine, C2-, C3-, and C4-carnitine. So even though acylcarnitine profiling can be considered a highly valuable diagnostic tool with regards to AM, it needs further validation and one should not blindly attribute a diagnosis of AM to a patient with increased acylcarnitines. It should be used as another diagnostic tool in combination with history, clinical signs, other laboratory findings, and histological examination.

Although great similarity exist between AM cases and human MADD cases when looking at the results of acylcarnitines, organic acids, and glycine conjugates, some differences should be acknowledged. Horses with AM show hyperglycemia [3, 10, 12, 16, 23, 24] and increased free serum carnitine levels [24], although a decrease in muscle free carnitine has been found [23], whereas humans with MADD have hypoglycemia and decreased free carnitine levels. These differences are possibly explained by species-specific steps in energy metabolism. In addition, humans have a deficiency of short-, medium-, and long-chain acyl-CoA dehydrogenases leading to a general accumulation of plasmatic acylcarnitines from C4 to C18, thus including long-chain acylcarnitines [40, 41]. The studies of Westermann et al. [24] demonstrated no significant increase in plasma and muscular long-chain acylcarnitines in affected horses (no or only very mild increases), and they only tested the muscle activity of short- and medium-chain acyl-CoA dehydrogenases, not of long-chain acyl-CoA dehydrogenases [24, 39]. More recently, cases have been noted to have significant elevations of long-chain acylcarnitines ([23]; unpublished results Votion et al.), suggesting also a deficiency in long-chain acyl-CoA dehydrogenases.

## 7. Postmortem Examination

At necropsy, the most consistent feature is the presence of dark-brown urine in the bladder [33]. Macroscopically visible muscular lesions are aspecific and consist of discolorations in postural and respiratory muscles, particularly intercostals, diaphragm, and muscles of the neck and shoulder. Pale areas may also be seen in the myocardium. However, in some cases, there is no lesion in either skeletal or cardiac muscles [10, 12, 16, 17, 22, 33].

## 8. Histology

Muscle biopsy for histological examination is best performed in living horses on postural muscles (more specifically the shoulder muscles) and at postmortem examination, on intercostals [33]. On histological examination of AM-affected muscles, severe lesions of Zenker's degeneration are visualized, which is characterized by loss of cross-striations, fiber swelling, hypereosinophilia of the sarcoplasm, hyaline degeneration, and necrosis. These lesions are multifocal, meaning that, on the same slide, affected and unaffected myofibers are visible. Lesions are severe in postural and respiratory muscles (i.e., rich in slow oxidative type I fibres) and may be absent or minimal in the locomotor muscles (i.e., rich in the glycolytic type II fibres) [17, 33]. There is no or minimal cellular response with infiltration by macrophages or more rarely neutrophils [10, 12, 13, 22, 33]. Neither glycogen not calcium salt accumulations are present in affected muscles, but a severe accumulation of lipids is demonstrated by Oil Red O (ORO) staining and electron microscopy [17, 22, 23, 33]. The presence of lipid vesicles is considered to have a high sensitivity and specificity for AM but is not a peculiarity of the condition and excessive intramuscular lipid storage may be found in other myopathies such as intoxication by ionophores (however, skeletal muscle lesions are poorly documented as opposed to lipid accumulation in the myocardium [42, 43]). In addition, seen the amount of myopathies in human beings caused by disorders in lipid metabolism [44] and the importance of fat substrates as energy source for the equine muscle [45], it is believed that other, yet undiscovered, myopathies may result in accumulation of lipid vesicles (more readily appreciated by transmission electron microscopy) within muscle fibers. Secondly, only limited information is available on the normal range of lipid storage in muscle samples stained with ORO from horses. One study has evaluated ORO staining with the help of a scoring system where a score of 1–3 was attributed for (1) the number of fibres containing lipid droplets, (2) the density of the lipid droplets within the fibres, (3) the size of the lipid droplets, leading to a final score of 0 and 9. A group of healthy horses had a maximum score of 6, suggesting a score of higher than 6 to be indicative of increased lipid storage. In a group of myopathic horses, a fourth of the cases had a score higher than 6, amongst which some but not all were AM cases [46]. In addition, hyperlipemic ponies have been described to have mild increased uptake in ORO staining in the muscle [47], and horses with AM frequently suffer from hyperlipemia [3]. These findings show the use of a scoring system for ORO evaluation to diagnose AM, but also its limitations.

## 9. Diagnostic Procedure

In summary, a diagnosis of AM should be based on the combination of history, clinical signs, and laboratory findings and histological examination [3, 10, 12, 13, 16]. Taking this principle into account, van Galen et al. [2] developed a decisional algorithm to obtain a clear case definition for epidemiological studies on AM, which allows to categorize cases that were reported to the AMAG as having AM or not. This algorithm can also be of diagnostic help to clinicians. However, even though that history, clinical signs, laboratory findings, and histological examination can be highly indicative, they are not pathognomic for AM. Therefore, this algorithm should in the future probably best be used as a first screening tool of suspected cases. As mentioned above, a more definitive diagnosis can be obtained by determination of acylcarnitine and organic acid profiles [23, 24, 39] but again it needs further validation with the suggested algorithm.

## 10. Prognosis

### 10.1. Survival

Survival rate was previously estimated from 3% [16] to 15% [14, 31], but possibility of survival was questioned [26]. During more recent European outbreaks (2006–2009) the overall survival rate was reported to be 26% (88/356 cases) and reached a maximum of 57% in France in 2006 [2]. The survivors in this study were mostly cases with a high suspicion of AM based on history, clinical signs, increase of serum CK activities, and presence of myoglobinuria, although some of them were confirmed with histology.

Most nonsurvivors died or were euthanized within 3 days of onset of clinical signs, however, some remained alive up to 10 days [3, 10, 12, 13, 16, 28, 31]. Surviving cases were recovered after 10.6 ± 5.6 days with a range of 1–30 days. Those where a followup was available appeared to recover uneventfully [22, 28]; however, only a minority have consistently been subjected to medical followup in order to exclude signs of subclinical disease. Recently one horse was described to still have arrhythmias after 10 weeks [34]. Caution is therefore required when discussing the long-term prognosis after a clinical episode of AM. Moreover, horses probably do not seem to develop protective immunity since they can get the disease a second time [2, 3].

### 10.2. Prognostic Factors

Indicators of prognostic factors have been recently identified by statistical comparison of variables of survivors *versus* those of nonsurvivors, by the calculation of sensitivity, specificity PPV, and NPV of clinical signs regarding survival status [38, 48]. All methods indicate a risk of survival or nonsurvival associated with certain variables, but this does not mean that survival (or mortality) is certain once a specific variable is present (or absent). Therefore, to implicate these prognostic indicators in a clinical setting, it is advised to evaluate the patient thoroughly, to make a prognostic assessment based on several variables and never on one solely, and to take into account the clinical evolution.

About clinical signs, nonsurvivors showed significantly more often recumbency, sweating, anorexia, tachycardia, tachypnoea, and dyspnoea compared to survivors, whereas defecation was significantly more often encountered in survivors. Remaining standing and normal mucous membranes had both a high specificity, PPV and NPV, and normothermia and defecation had both a high sensitivity and NPV when they were used to predict survival [48]. Regarding to the high specificity, PPV, NPV, and sensitivity this means, respectively, that

- the proportion of nonsurviving AM cases without ‘‘remaining standing” or ‘‘normal mucous membranes” on the total of nonsurviving AM cases is high (few nonsurvivors have these signs);
- the proportion of AM survivors with ‘‘remaining standing” or ‘‘normal mucous membranes” on the total of AM horses with these signs is high (few AM-affected horses with these signs are nonsurvivors);
- the proportion of AM nonsurvivors without ‘‘remaining standing” or ‘‘normal mucous membranes” or ‘‘normothermia” or ‘‘defecation” on the total of AM horses without these signs is high (few AM-affected horses without these signs are survivors);
- the proportion of AM survivors with ‘‘normothermia” or ‘‘defecation” on the total of AM survivors is high (most survivors show these signs).

As mentioned above, it should be pointed out that PPV and NPV are related to the prevalence of the condition in the studied population [37], and especially those variables with a high sensitivity (i.e., normothermia and defecation) are most useful as screening tool to identify survivors.

Even though serum CK activity can be highly interesting to monitor on-going muscle damage, serum CK activity (at first examination and/or the highest recorded value) was repeatedly found not to be higher in worse cases [2, 3, 38]. Also, serum cardiac troponin I was not correlated with survival [34]. Other blood parameters were, however, found to have prognostic value by a study on 34 hospitalised cases on admission [38]. This study has limited statistical strength due to low case numbers and therefore results should be interpreted carefully. Nevertheless, these results can be of aid to clinicians when combined with the above-mentioned clinical prognostic indicators. Nonsurvivors were shown to have a significantly higher PCV compared to survivors. In addition, analysis showed that the base excess based on plasma chloride concentrations (BEcl), plasma chloride concentrations, and PCV had strong discriminatory power between survivors and nonsurvivors, with nonsurvivors having a higher BEcl and PCV, and lower chloride levels. The variable BEcl was calculated in this study by the following equation:

(1) BEcl=Cl− normal−(Cl− measured×Na+ normalNa+ measured),(Na+ normal=140 mmol/L,  Cl− normal=105).

Moreover, the same analysis allowed development of an optimal tree to identify nonsurvivors based on BEcl, urea, and heart rate with a sensitivity and specificity of 84% and 93%, respectively. It concludes that cases with the following three criteria were likely to end as nonsurvivors: BEcl of higher than 3.32, a urea higher than 5.26 mmol/L, and a heart rate of higher than 40 bpm.

Respiratory distress and/or failure often develops in affected cases and can be demonstrated by a low partial pressure of arterial oxygen (PaO_2_), hypercapnia (venous partial pressure of carbon dioxide >53 mmHg). Based on clinical evolution of nonsurvivors, the occurrence of hypoxemia (<60 mmHg) and hypercapnia was suggested to be associated with mortality [3, 38].

Even if certain clinical signs and blood parameters were identified as having prognostic value, it still remains difficult to recognise horses that might survive a clinical episode of AM. Moreover, the clinical status of AM patients can deteriorate, but also improves rapidly (personal observation).

## 11. Treatment and Management of Affected Horses

As long as the causative agent is not identified with certainty, treatment remains symptomatic and supportive. Nevertheless, treatment can be extrapolated from and based on the described clinical signs and current pathophysiological knowledge of the condition, more specifically the identification of a MADD-like syndrome [24] and decreased activity of mitochondrial respiratory chain complexes [17, 33, 49]. Because FAD is cofactor of the deficient mitochondrial dehydrogenases in MADD and because riboflavin is a precursor of FAD, a riboflavin deficiency was considered [24]. However, plasma riboflavin concentrations of AM-affected horses did not differ from those of healthy controls, excluding a deficiency but leaving the possibility of a competition or blockage by some yet unidentified toxin [24]. The occurrence of lactic acidosis and respiratory alkalosis in affected horses [38] is possibly directly related to anaerobic metabolism.

The following actions and treatments can be considered for horses that suffer from AM:

- horses should be stabled as soon as possible before recumbency and hypothermia (i.e., favoured by recumbency in a cold environment) occur [2, 3, 13, 16], and to remove them from the “toxic” pasture;
- in order to avoid or limit a negative energetic balance and respiratory alkalosis, it is probably indicated to avoid physical effort and stress as much as possible;
- horses should be kept as quiet and comfortable as possible with the aid of muscle relaxants and if needed nonsteroidal anti-inflammatory drugs or other analgesics;
- individually adapted fluid therapy is recommended in order to restore circulating volume, promote myoglobin excretion, and correct acid-base and electrolyte imbalances and should be individually tailored;
- digestive, respiratory, cardiac, and urinary functions warrant close monitoring and support [2]. Considering the metabolic problems, support of aerobic mitochondrial metabolism and favouring carbohydrate metabolism over lipid metabolism seem reasonable [22, 24]. This can be done by restoring circulatory volume, providing further cardiorespiratory support, feeding a fractioned carbohydrate-rich diet, and administering intravenous glucose solutions;
- B vitamins, including riboflavin, are important for aerobic metabolism and although they are usually sufficiently provided in good quality roughage [50], it can be that affected horses are in need of bigger amounts. Supplementation might avoid them from becoming a limiting step and is therefore advised;
- carnitine supplementation can be considered because it might improve lipid metabolism, even though free carnitine levels were shown to be already elevated in blood in affected horses [24]. In human beings with defects of *β*-oxidation enzymes, however, there is no objective evidence of its beneficial effects, and it may even be arrhythmogenic in long-chain defects [44]. Currently, it is not known if carnitine causes negative side effects in horses with AM;
- it is worth noting that the therapeutic group consisting of vitamins, antioxidants (vitamins B, C, E, and selenium), and carnitine was the only medical support that appeared to be more often used in survivors compared to nonsurvivors and can thus be considered beneficial for outcome [28]. Also the authors of the American case series suggested antioxidant therapy (vitamin C, vitamin E, and selenium) [22, 51]. In addition, an *in vitro* study has demonstrated that cells suffering from short-chain acyl-CoA dehydrogenase deficiency (a part of the complex biochemical problems in AM) are vulnerable to oxidative stress and incubation with antioxidants significantly increased their viability [52]. These findings highlight the importance of these compounds for the treatment of AM, but it does not mean that other therapies are not useful;
- dantrolene is probably not indicated for AM-affected horses since important accumulations of calcium have not been visualised in affected muscle samples [33], and more importantly dantrolene has an inhibitory effect on complex I of the respiratory chain [53].

All the above-mentioned treatments are only suggestive, and no or only limited direct scientific proofs of true efficacy of these treatments, drugs, or supplements on AM are available and therefore further investigation is necessary. For detailed suggestions according to management of AM patients, the reader is referred to [54, 55].

## 12. Management of Pasture Companions

When AM declares on a pasture, it is strongly advised to check the seemingly healthy pasture companions of the affected horse. These pasture companions most likely have also been in contact with the causative agent and are often submitted to the same predisposing and/or contributing factors as their sick friend. They are thus at risk to develop the disease in the following hours or days, or they may suffer already subclinically from the disease [3, 13].

Without creating stress for these pasture companions, preventive measures as discussed in the following chapter should be applied to them, even if this might sound like “last minute” prevention. More specifically, pasture companions should be removed from the pasture; ideally they should be stabled full time, at least during the risk period. Stress, such as transport, anaesthesia, or exercise, should be avoided as much as possible to limit muscular energetic deficits that favour clinical disease. Without excess, they should receive supplementary food, by preference carbohydrate rich food. These pasture companions need also close clinical surveillance and serum CK activity should regularly be measured [2]. As soon as serum CK activity is increased or if clinical signs are noted, the horse should receive supportive treatment to limit any further muscle damage. Vitamins and antioxidants can be administered as preventive therapy to support muscle mitochondrial metabolism, moreover since they seem to have a positive effect on outcome in clinical cases [28].

## 13. Prevention of Atypical Myopathy

Since supportive therapy is still often unsuccessful, prevention of this severe condition is of major importance [26]. Preventive measures are based on descriptive data, but also on risk and protective factors. Risk and protective factors were determined by statistical analysis of data of affected horses compared to those of a control group. If a certain parameter occurs more frequently in the group of affected horses compared to a control group this parameter is determined as a risk factor for AM. The opposite, a parameter occurring more frequently in a control group *versus* the affected group, is considered a protective factor for AM. The determination of risk and protective factors is a statistical risk analysis, which means that the risk for a horse to develop AM increases, but is not certain, when risk factors are present or protective factors are absent, and that the risk decreases, but is not zero, when protective factors are present or risk factors are absent. Therefore, AM still can occur in absence of risk factors or in the presence of protective factors.

### 13.1. Preventive Measures Based on Risk and Protective Factors

A first risk analysis was performed on 57 Belgian nonsurviving cases from 2000 to 2005. They were compared to clinically healthy cograzers and to healthy grazing control horses that were matched for the region where AM broke out [3, 29]. A second analysis was performed on 354 European cases that were highly suspicious or confirmed to have AM from 2006 to 2009, including survivors. They were compared to cases that were reported to suffer from AM, but finally had another diagnosis or had a low probability to suffer from AM.  Table 3 summarizes the risk and protective factors drawn from these studies and Table 4 lists the preventive measures that can be concluded from these risk factors and descriptive data.

#### 13.1.1. Demographic Data

Whenever possible, preventive measures should be applied to all horses, but if a choice should be made between horses due to limitations of infrastructure or management, horses at greater risk should be favoured. Young horses have been demonstrated to be at risk to develop AM. Young horses spend probably more time at pasture and are less frequently submitted to intensive training compared to older horses. Not being intensively trained seems to favour the development of AM. In addition, in a group of unexercised Standardbred mares at pasture, young horses have been demonstrated to have significantly more type I myofibers compared to old horses [56], therefore having a more important amount of target myofibers for the causative agent of AM. Besides age, also body condition seems to influence the development of AM, which might be explained by the capacity or not to compensate for the occurring energetic imbalance with the aid of body reserves.

#### 13.1.2. Horse Management

Training is considered a protective factor, and most affected horses were in no or only limited training at the time of diagnosis [2, 3, 10, 12]. Training increases oxidative capacity [57, 58] and horses with intense training schedules often receive supplementary feedings and spend less time at pasture. Moreover, horses are only used intensively for training from a certain age. To reduce the risk for AM, it can be advised to regularly exercise grazing horses and to favour nonexercised horses for other preventive measures. However, if the horse suffers already from (sub)clinical disease it is contraindicated to work the horse.

Since it is thought that horses get in contact with the causative agent while being at pasture, preventive measures limiting pasturing are of major importance to prevent AM. During high-risk seasons and/or during outbreaks it is advised to stable horses and avoid pasture. If stabling is, however, difficult or impossible or if stabling is considered no good practice for horse welfare, pasturing should be limited. Less than 6 hours a day or weather-dependant pasturings are considered protective and are suggested as valuable alternatives.

Even though it is unexpected that the causative agent directly originates from the drinking water, water might have an indirect influence on the development of AM. It has been shown that providing water from the distribution network and in a tank or a bath are protective factors. This might avoid that horses go to and drink from water courses and streams and that they spend time in humid zones. Humidity at pasture has been determined as a risk factor and might provide the causative agent with good conditions to exert its toxicity.

Providing supplementary feeding and a salt block were determined as protective factors. They should be provided all year round, but especially during high-risk periods. Due to supplementary feeding and a salt block horses are probably less prone to eat substances that they would normally not consume when sufficient nutrients are available; they might be more selective in grazing, and/or graze less. Moreover, it provides the horse with protective antioxidants, vitamins, and minerals that might counteract on the metabolic imbalance and support muscle function. Supplementary feeding can consist of silage, straw, complete mix, corn, or oats. However, one must be careful with giving hay in the autumn [29]. Hay is often given from the ground and thus possibly favours contact with the causative agent.

#### 13.1.3. Pasture Characteristics and Management

Pastures with a history of previous dead horses, regardless the cause and year, were found to be risk factors for AM and should be avoided whenever possible. In addition, pastures where AM has declared in the past remain a danger for horses grazing on it, as pastures have been described to declare AM in successive years [2, 3].

Pastures that are lush during the winter season were found to be risk factors for AM. These data should be interpreted with caution, because a discrepancy between observations by scientists and horse owners was noted. Owners often described their pastures as having lush grass, but regularly scientists found them to be bare [29].

Sloping pastures, especially those with a steep slope, should be avoided. This can be a confounding effect with the geographic area where AM regularly occurs, but it might in one way or another also play a role in creating an optimal microclimate for the causative agent. Horses grazing on sloping pastures probably also use their postural muscles more intensively and this might add to the energetic imbalance when muscles are affected.

Pastures that contain dead leaves and dead wood form a risk and therefore it is advised to remove dead leaves and wood from the pasture and burn them. Pastures surrounded by or containing trees were also determined to be risk factors, and almost for all described cases in the studies of Votion et al. [29] and van Galen et al. [2] (97 and 98%, resp.) trees were present at pasture. Trees, dead leaves, and dead wood might contain the causative agent, favour its development, and/or contribute to its toxicity or act in synergy with it.

### 13.2. Preventive Measures Based on Descriptive Data on AM

#### 13.2.1. Geographic Zones

Most of western European countries have had cases suffering from AM, but Belgium (especially the Walloon region), France (especially the north east regions), and Germany (especially the mid-west regions) are known to have had multiple large outbreaks [2, 3, 13, 16, 31]. These countries or areas probably share a similar landscape, flora and/or management of pastures and horses that combine multiple risk factors during high risk periods.

#### 13.2.2. Season

High-risk seasons are defined as autumn and spring, but cases do not declare every year. Winter cases are mostly cases occurring during early winter just following autumn, or late winter just before spring. After an outbreak in autumn, some cases can be expected to occur in the following spring [2].

#### 13.2.3. Climatic Conditions

As previously mentioned, outbreaks cease after several days of heavy frost and/or when the pastures are covered with snow (personal observation). Nevertheless, the occurrence of AM remains unpredictable and one should not solely rely on weather forecasts to decide whether horses should be submitted to preventive measures for AM. However, during ongoing outbreaks, it is advised to reduce the pasturing time when the weather is inclement.

#### 13.2.4. Demographic Data

Even though that some horses are more at risk than others as discussed in the previous chapter, all equids (all horse breeds as well as donkeys and zebras, both sexes, all ages, and thin, normal, and obese horses) can be affected by AM [2] so preventive measures should ideally be applied to all types of equids.

#### 13.2.5. Horse Management

Since AM affects severely the horse's energy metabolism, it can be suggested to fraction feedings and to favour a carbohydrate rich diet (concentrates, good quality of fibres) over a diet rich in fat in order to optimally support the energy and nutrient supply. Limiting pasturing has been defined as a protective factor, but above this, AM has not been described in horses spending less than 6 hours at pasture [2]. This suggests that contact time with the pasture is of major importance to develop the disease.

#### 13.2.6. Pastures

Often AM declares on natural and untreated pastures [29]. A general aspecific treatment of pastures can therefore be suggested. Ploughing, sowing, mowing, and fertilizing, for example, influence the soil and plant cover and might interfere with the causative agent and/or predisposing factors.

Although the presence of trees was a risk factor for the development of AM, tree species have not been evaluated by statistical risk analysis. Nevertheless, one tree in particular has been mentioned repeatedly in relation to AM: *Acer pseudoplatanus* (maple tree) [3, 32]. It is unknown for the time being if this common European tree plays a role in the pathogenesis of AM. It is strongly advised to avoid using pastures surrounded by or containing trees during risk seasons, and this measure counts especially for the maple tree.

## 14. Etiological Hypotheses

Over the history of AM, several causes have been proposed, some of which have been investigated. Toxic products, such as ionophores, herbicides, weed killers, nitrates, and nitrites, have been incriminated but searching for these products gave negative results [12, 16]. In addition, epidemiological studies have discarded accidental contamination of horse feed by ionophores during its industrial production or an accidental access to ruminant foodstuffs: none of the cases reported to AMAG had access to such feed. Also, acute intoxication due to pollutants is unlikely since the large majority of pastures were untreated and natural [29].

Selenium or vitamin E deficiency can lead to a nutritional myopathy, often resembling AM [14]. Antioxidant status of AM cases was variable [10, 12, 16] and nutritional myopathy is not believed to be the cause of AM. However, as mentioned above, these important antioxidants were the only medical support that appeared to be beneficial for a positive outcome [28].

The seasonality of AM and its link with climatic conditions that are favorable to fungal growth have raised the hypothesis of the action of a mycotoxin. *Fusarium* fungi [16], *Trichoderma* fungi [1], and *Stachybotrys chartarum* (unpublished data) were found in some grass samples collected on affected premises, but not consistently. All of them can produce trichothecene mycotoxins, but those toxins are not known to induce rhabdomyolysis syndrome. In one study, *Acremonium lolii*, a fungal endophyte known to produce toxins called lolitrems, was found in grass samples [16]. However, in the ryegrass staggers syndrome disease induced by lolitrem B poisoning no rhabdomyolysis is induced [59] and, moreover, lolitrem B was not found following analysis of the samples from AM cases.

The presence of *Clostridium sordellii* lethal toxin in muscle of AM-affected horses suggests a possible role of this toxin as a trigger or even as lethal factor in this disease [60]. Further evidence is needed before we can consider that this bacterium is implicated in the disease. If it is, the development of a protective vaccine still remains hypothetical since naturally affected horses do not mount a protective immune response [2, 3].

Plants known to be toxic for horses or other animals were not consistently present in the pastures of affected horses and/or were previously identified to cause other clinical signs than rhabdomyolysis [3, 10, 14, 16, 31]. However, the possible role of maple leaves contaminated with the endophyte *Rhytisma acerinum* (European tear spot) in the etiology of AM has been suggested [32] if even, no scientific data supports a link with this fungal leaves disease and the identified MADD syndrome.

A much more interesting hypothesis about the etiology of AM has been raised by scientists at the University of Minnesota Equine Center that have found the toxic hypoglycin A metabolite, methylenecyclopropylacetic acid (MCPA) in conjugated form in SPM horse serum and urine [61]. Hypoglycin A may be found in seeds of several tree species including members of the genus *Acer* [62] and is known to induce, *in vivo* and *in vitro*, inhibition of several FAD dependant mitochondrial dehydrogenases causing widespread disturbances of the oxidation of fatty acids and several amino acids [63–65]. Currently, sera collected on European AM cases are being analyzed in collaboration with the University of Minnesota to search for MCPA conjugates in blood samples from European AM cases. On a positive note, this toxin in North America could very well be the cause of AM in Europe; that is, hypoglycin may be contained in seeds of *Acer* spp. trees on European pastures where AM has occurred.

## 15. Conclusion

Atypical myopathy is an emerging condition in Europe. It is highly fatal, but survival is possible. This points out the necessity of a rapid and accurate diagnosis, so that supportive treatment can be started as soon as possible. A prognostic assessment of the patient can help to decide whether treatment is justified. Treatment is indeed often unsuccessful and preventive measures are therefore of major importance. Further research is needed on this intriguing condition to further improve diagnosis, treatment, and prevention. The development of AM is probably multifactorial with important environmental components that deserve further investigations.

## Figures and Tables

**Table 1:** European cases communicated to the Atypical Myopathy Alert Group from autumn 2006 up to spring 2012.

Reported cases	Total 2006–2012	Autumn 2006	Spring 2007	Autumn 2007	Spring 2008	Autumn 2008	Spring 2009	Autumn 2009	Spring 2010	Autumn 2010	Spring 2011	Autumn 2011	Spring 2012
Austria	3											3	
France	393	29	1	11	17	10		127	103	29	7	41	18
Germany	187	7		3	5			93	19	1		57	2
Belgium	191	46	7	18	6	6		66	10	14	2	16	
Denmark	5	3						2					
Luxembourg	3	1						2					
Ireland	2							2					
The Netherlands	59	13		3		2		34	6	1			
United Kingdom	132	1		14				39	17	22		35	4
Switzerland	50			9				30	3	1		7	
Spain	32									1		31	
Czech Republic	1									1			
Unknown	2								1			1	

Total	1060	100	8	58	28	18	0	395	159	70	9	191	24

Comments: September or early winter was also considered autumn; early summer was considered spring. This is of concern for less than 10% of cases.

**Table 2:** The frequency of the main clinical features of AM as described by Belgian [3], French [31], and European [2] epidemiological studies.

	France (*n* = 82) 2002-2003 (%)	Belgium (*n* = 57) 2000–2005 (%)	Europe (*n* = 354) 2006–2009 (%)
Depression	74	72	80
Weakness	Not mentioned	100	85
Stiffness	82	80	83
Recumbency	90	96	78
Trembling	Frequent	71	68
Sweating	58	57	64
Pigmenturia	100	95	93
Distended bladder on rectal palpation	Not mentioned	80	58
Congested mucous membranes	Not mentioned	69	53
Tachycardia	76	69	79
Dyspnea	70	68	44
Normothermia	48	36	60

**Table 3:** Risk and protective factors for the development of atypical myopathy.

	Risk factors	Protective factors
Demographic data		
Age	Young horses (<3 years)	
Sex*	(i) Colts (ii) Stallions	Geldings
Body condition	(i) Thin (ii) Normal weight	(i) Overweight

Management practices at the horse level		
Deworming		Frequent deworming
Vaccination		Regular vaccination
Occupation		Training
Pasturing	Full-time pasturing (all year round)	(i) Not at pasture (ii) <6 H per day at pasture (iii) Weather-dependant pasturing in spring and in autumn

Food and water		
Supplementary feeds	Hay in autumn	(i) Supplementary feeds in autumn (except hay), in particular silage and complete mix (ii) Supplementary feeds in winter, in particular straw, silage, complete mix, and corn (iii) Supplementary feeds in spring (iv) Supplementary feeds in summer (v) Salt block (all year round)
Water supply		(i) Distribution network (ii) In tank or bath tub

Pasture characteristics		
History	Dead horses on the pasture in the past	
Grass land	Lush pasture in winter	
Incline	(i) Sloping pasture (ii) Steep slope	Gentle slope
Trees	(i) Surrounded by or containing trees (ii) Presence of dead leaves (iii) Presence of dead wood	
Humidity	(i) Humid pasture (ii) Pasture surrounded by or containing a stream/river	

Management of the pastures		
	Spreading of manure	

*Age was identified as a confounding variable regarding the risk factors associated with the animal's sex. Sex is linked to age, as “gelding” is a status that follows castration, a procedure usually performed in males >18 months old (i.e., the most risky age for AM). Because females are more frequently kept at pasture than males, a higher percentage of AM cases is females.

**Table 4:** Summary of preventive measures for the development of atypical myopathy.

Where?	
All over Europe, but especially in Belgium, France, and Germany	
When?	
(i) High-risk seasons (mainly autumn and spring)	
(ii) Spring after an autumnal outbreak	
(iii) During outbreaks	
During which weather conditions?	
(i) Lack of solar radiation	
(ii) Strong wind	
(iii) Rain and thunderstorms	
(iv) Cool temperature without heavy frost	
For how long?	
Until 4-5 days of daytime frost or snow	
For all grazing equids, but horses particularly at risk are	
(i) Young horses	
(ii) Horses with normal body condition	
(iii) Untrained horses	
Horse management	
(i) Regular deworming and vaccination	
(ii) Provide supplementary feeding	
(iii) Do not feed hay from the ground	
(iv) Provide water from the distribution network and in a tank or a bath	
(v) Regular exercise	
(vi) Stable horses or limit pasturing during the risky seasons (<6 H a day or weather-dependant pasturing)	
(vii) Provide a salt block	
Pasture management	
(i) Avoid spreading of manure on the pasture; prefer manual removal of faeces	
(ii) Avoid pastures where previous deaths have occurred	
(iii) Dead leaves and wood should be removed from the pasture and be burned	
(iv) Ensure rotation of pastures and avoid (during the risky seasons) (1) sloping pastures, especially those with a steep slope, (2) bare pastures, (3) humid pastures or pastures surrounded by or containing a stream or river, and (4) pastures surrounded by or containing trees	
(v) Provide general aspecific pasture treatment such as ploughing, sowing, mowing, and fertilizing	
